# Carbamazepine-Induced Systemic Lupus Erythematosus in a Patient With Idiopathic Trigeminal Neuralgia: A Case Report

**DOI:** 10.7759/cureus.47009

**Published:** 2023-10-14

**Authors:** Hidetaka Kuroda, Kentaro Taniguchi, Shota Tsukimoto, Uno Imaizumi, Motohiro Komaki, Takuro Sanuki

**Affiliations:** 1 Department of Dental Anesthesiology, Kanagawa Dental University, Yokosuka, JPN; 2 Department of Periodontology, Kanagawa Dental University, Yokosuka, JPN

**Keywords:** trigeminal neuralgia, systemic lupus erythematosus, idiopathic trigeminal neuralgia, drug-induced lupus erythematosus, carbamazepine

## Abstract

A 50-year-old woman presented with a mandibular second molar and facial pain and was diagnosed with idiopathic trigeminal neuralgia. Carbamazepine (CBZ) was initiated at 300 mg/day, successfully relieving the pain. However, on the 8th day of CBZ treatment, the patient developed symptoms resembling those of systemic lupus erythematosus with malaise, nausea, and facial erythema. CBZ was immediately discontinued. Subsequently, she experienced numbness in both lower limbs and mild fever, which resolved within a few days. Laboratory tests revealed leukopenia (2.8 × 10^3^/μL), elevated C-reactive protein levels (0.46 mg/dL), and the presence of antinuclear antibodies (ANA) and anti-Sjögren's syndrome-related antigen A antibodies. The clinical course suggested CBZ-induced drug-induced lupus erythematosus (DILE). This case highlights the possibility of DILE onset even after short-term CBZ treatment, the importance of prompt discontinuation of the causative drug in patients suspected of DILE, and the conduct of ANA testing in diagnosing DILE.

## Introduction

Drug-induced lupus erythematosus (DILE) is an autoimmune disease triggered by the long-term use of medications that produce symptoms and laboratory findings similar to those of systemic lupus erythematosus (SLE). The general symptoms of SLE include joint pain and arthritis, malar rash and other skin rashes, pleuritis or pericarditis, kidney or central nervous system involvement, and hematologic cell count reduction [1]. The major signs and symptoms include joint pain/arthritis, rash, fever/fatigue, pleuritis/pericarditis, lymphadenopathy, and oral or nasal ulcers [2]. Although pulmonary and pleural lesions can complicate SLE, DILE is often underestimated owing to its mild disease course. Therefore, DILE is rarely diagnosed correctly and accounts for only 10% of all SLE cases [3]. Approximately 80 drugs have been implicated in the occurrence of SLE-like reactions or the exacerbation of pre-existing SLE, including antiarrhythmic drugs, antihypertensive drugs, antipsychotics, antibiotics, anticonvulsants, antithyroid drugs, anti-inflammatory drugs, diuretics, cholesterol-lowering drugs (statins), and biological agents. Of these drugs, hydralazine, procainamide, and quinidine have the highest incidence of causing SLE. However, the antiepileptic drug carbamazepine (CBZ) rarely induces DILE [4,5]. A literature review of 27 patients with CBZ-induced SLE (CBZ-DILE) reported the accidental development of the aforementioned disease after medication use for more than a year [2]. In this case report, we describe a case of a patient with idiopathic trigeminal neuralgia who developed CBZ-DILE following a short course of oral medication. Written informed consent was obtained from the patient.

## Case presentation

**Figure 1:**
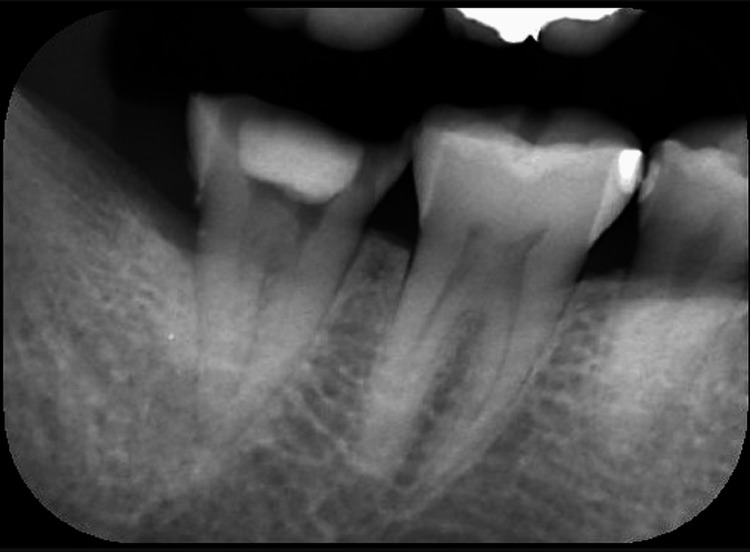
Intraoral radiograph In the right mandibular second molar, a root canal treatment was in progress, but there were no findings that would cause throbbing pain.

A 50-year-old woman complained of throbbing pain in the right mandibular second molar. The tooth was subjected to root canal treatment. Pain was induced during eating, speaking, and tooth brushing and was rated as 10 on the numerical rating scale. Periapical, occlusal, or apical swelling and pressure pain on the tooth were not noted. Intraoral radiography showed the absence of abnormalities (Figure 1). There was no evidence of periodontal disease around the tooth, and compression of the right masseter muscle did not induce toothache.

**Figure 2:**
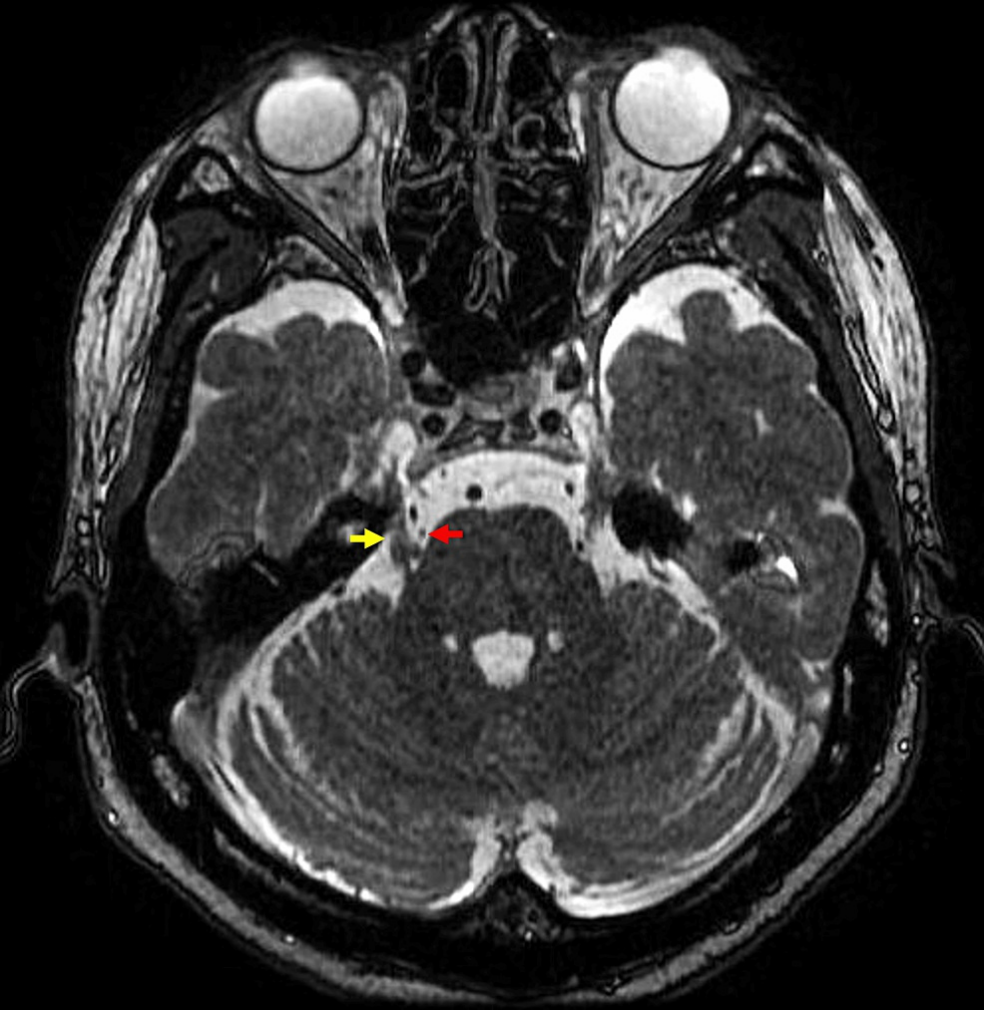
Horizontal section of MRI The arrowheads showed proximity of the superior cerebellar artery (red) and trigeminal nerve (yellow), but there was no evidence of neurovascular compression.

Along with toothache, the patient complained of ipsilateral electric shock-like pain after washing his right mandible. The pain lasted for a few seconds. The patient had no underlying diseases. Neurovascular compression, multiple sclerosis, and other space-occupying lesions were not noted on magnetic resonance imaging (MRI) (Figure 2). Based on the pain characteristics and MRI findings, the toothache and facial pain were due to idiopathic trigeminal neuralgia, purely paroxysmal (International Classification of Orofacial Pain, 4.1.1.3.1) [6], and a CBZ dose of 300 mg/day was administered.

A day after taking CBZ, the toothache and facial pain disappeared; however, the patient experienced general malaise, nausea, discomfort, and erythema on both sides of the cheek (lupus) on the 8th day after initiating the medication. Hence, the patient was immediately instructed to discontinue medication. On the second day after the onset of illness, nausea and discomfort persisted, while numbness in both lower limbs and a slight fever (37.2°C) developed. Screening laboratory test revealed a white blood cell level of 2.8 × 103/μL (normal: 3.3-8.6 × 103/μL) and a C-reactive protein (CRP) level of 0.46 mg/dL (normal: less than 0.15 mg/dL). On the third day, the fever, nausea, and dysphoria resolved; however, numbness and lupus persisted in both lower limbs. On the 4th day, the numbness in both lower limbs and erythema on the face decreased, and the symptoms completely disappeared on the 5th day.

One month later, the patient was referred to a rheumatologist. Biological laboratory tests showed a normal white blood cell count and fraction level, a serum IgG level of 2,042 mg/dL (normal: 870-1,700 mg/dL), and a rheumatoid factor level of 98.2 IU/mL (normal: <15 IU/mL). The patient tested positive for nucleolar and cytoplasmic antinuclear antibodies (ANA) and anti-Sjögren's syndrome-related antigen A (anti-SSA) antibodies.

## Discussion

The present case report documented the occurrence of SLE-like symptoms after a short course of CBZ administration.

DILE is generally diagnosed based on the following criteria: (1) the absence of lupus symptoms prior to the initiation of therapy; (2) the presence of one or more clinical signs of SLE and characteristic immunological abnormalities; and (3) the resolution of these signs upon discontinuation of therapy [2,7]. In the present case report, the patient had no signs of SLE prior to CBZ administration; general malaise, lupus, numbness in both lower limbs, and low-grade fever only occurred after the initiation of treatment, which disappeared after discontinuing CBZ. Moreover, the immunological test showed positive ANA, thus suggesting that the patient had possibly developed CBZ-DILE. The mechanism of SLE onset due to antiepileptic drugs involves two potential pathways: antiepileptic drugs acting as initiators by inducing the production of anti-DNA antibodies in SLE-like DNA carriers and antiepileptic drugs inducing SLE, causing SLE to develop endogenously even in the absence of significant symptoms before administration [7]. However, the pathophysiological mechanism that triggered the occurrence of SLE in this patient remains unclear.

The thresholds for onset based on the dosage, incubation period, and duration required for recovery in CBZ-DILE vary. A previous literature review reported that CBZ-DILE occurred after a latency period of 36.5 ± 60.2 months with daily oral doses ranging from 200 to 1600 mg/day, and the duration of recovery after discontinuation of the drug ranged from one week to 18 months [2]. In the present patient, the symptoms developed a week after the oral administration of CBZ; hence, the medication was promptly discontinued. This finding suggests that the symptoms were very mild and lasted only for a few days. DILE can be adequately treated by early disease detection and withdrawal of the causative agent; however, in patients with refractory cases, corticosteroids, antimalarial drugs, and immunosuppressive agents are used in combination [2]. In this case, the rapid resolution of symptoms was observed, and pharmacological therapy for CBZ-DILE was deemed unnecessary. Differential diagnosis for DILE involves considering idiopathic SLE and idiopathic subacute cutaneous lupus erythematosus (SCLE). Some patients with serologic and clinical findings typically associated with these conditions might have DILE [3]. DILE is typically diagnosed by a process of elimination, as it is characterized by an improvement in symptoms when the causative drug or agent is withdrawn in patients who previously had a normal immune system. Therefore, ruling out SLE or SCLE is often a crucial step in diagnosing DILE.

A limitation of this case study is the delayed administration of ANA testing. In most patients with CBZ-DILE, the ANA tests show a positive result with a homogeneous staining pattern and the presence of anti-histone and anti-double-stranded deoxyribonucleic acid antibodies [2,5]. In the present patient, one month later, the laboratory tests showed the presence of both ANA and anti-SSA antibodies, thus suggesting Sjögren’s syndrome. However, considering that the patient with CBZ-DILE tested positive for anti-SSA antibodies [8], this finding does not negate the diagnosis of CBZ-DILE in this patient. Although prompt ANA testing might have yielded different findings, the observation of leukopenia, elevated CRP level, and the nucleolar pattern of ANA in this patient could have provided supportive data for the diagnosis of CBZ-DILE [5,9,10]. It has been reported that in 40% of CBZ-DILE patients, the ANA had reverted to negative status within a timeframe ranging from three to 30 months. In the remaining 60%, the ANA remained positive throughout the variable follow-up period, typically at lower titers, with some specificities eventually becoming negative [2,8]. A follow-up ANA testing may be advisable in this case.

## Conclusions

In conclusion, CBZ can induce DILE shortly after the initiation of medication. When DILE is suspected, prompt discontinuation of the causative drug is crucial. Prompt ANA testing may be effective in diagnosing CBZ-DILE.
